# A novel clinically-oriented classification of fine-needle aspiration cytology for salivary gland tumors: a 20-year retrospective analysis of 1175 patients

**DOI:** 10.1007/s10147-020-01816-5

**Published:** 2020-11-20

**Authors:** Masataka Taniuchi, Ryo Kawata, Shuji Omura, Shin- Ichi Haginomori, Tetsuya Terada, Masaaki Higashino, Yoshitaka Kurisu, Yoshinobu Hirose

**Affiliations:** 1 Department of Otorhinolaryngology, Head and Neck Surgery, Osaka Medical College, 2-7 Daigaku-Machi, Takatsuki, Osaka 569-8686 Japan; 2 Department of Pathology, Osaka Medical College, Takatsuki, Osaka Japan

**Keywords:** Classification, Fine-needle aspiration, Cytopathology, Parotid tumor, Histological grade

## Abstract

**Background:**

When determining treatment strategy for a salivary gland tumor, assessing histology and malignancy grade before surgery is essential. Several new diagnostic classification systems for salivary gland cytology have recently been proposed. However, none incorporate histology and grade of malignancy.

**Methods:**

We developed a new cytology classification system that incorporates histology and grade of malignancy of salivary gland tumors (OMC classification), consisting of 11 categories. Our OMC classification was applied to 1175 patients who had preoperative cytology and confirmed final pathological diagnosis available from the past 20 years at our hospital (benign tumor: 981 patients, malignant tumor: 194 patients).

**Results:**

Based on the cytology, 729 patients (62.0%) had benign histology (Category 4–1), and 87 patients (7.4%) were diagnosed with grade of malignancy (Category 6–3 + 6–4). Based on the final pathological diagnosis, the accuracy rate of Category 4–1 and Category 6–3 + 6–4 of our classification system was 93.4% and 88.5%, respectively.

**Conclusion:**

Based on the correct diagnosis rate, the inclusion of histology and grade of malignancy in the salivary gland cytology classification was considered feasible. Thus, the OMC classification system is considered a useful tool when determining the treatment strategy for a salivary gland tumor.

## Introduction

Fine-needle aspiration cytology (FNAC) is a well-established procedure for the diagnosis of salivary gland tumors. Preoperative diagnosis with FNAC can help determine the extent of required resection, the planning for neck dissection, and the preservation of the facial nerve, as well as help in counseling patients with a parotid tumor [1, 2]. Moreover, this is particularly important because the management of parotid lesions depends on whether the tumor is benign or malignant, the histological type, and the histological grade of malignancy.[3]. Therefore, even if a surgical procedure is considered appropriate, the histology and grade of malignancy should be obtained before the initial operation, especially in the case of malignant tumors.

The Bethesda system for reporting thyroid cytopathology is commonly used for cytology reporting of thyroid glands [4]. Recently, a group of international pathologists collaborated and proposed a uniform reporting system, known as the Milan reporting system for salivary gland cytopathology [5]. The Milan system consists of seven categories, including nondiagnostic cases. For tumor lesions, an emphasis is placed on whether they are benign or malignant. However, when considering FNAC as part of the preoperative diagnosis, a reporting system should be useful for determining the treatment strategy, especially to assist in determining and planning surgical procedures. Although the Milan System does not recommend that a report should consist solely of the category number, optional reports including histopathology and the grade of malignancy is left to the individual pathologist or laboratory in benign and malignant tumors. In short, for benign tumors, a histological diagnosis, such as pleomorphic adenoma and Warthin tumor, is important. Enucleation needs to be avoided for pleomorphic adenoma, whereas continuous monitoring is an option for a Warthin tumor [6]. For malignant tumors, grade of malignancy is important, as the prognosis is markedly different between low/intermediate-grade and high-grade malignancy. Moreover, surgical approaches are different, including the preservation of the facial nerve [7]. Accordingly, as histopathological types vary among salivary gland tumors, a clinically-oriented classification of FNAC is warranted. In this classification, we try to describe histopathology and the grade of malignancy as definite as possible.

The objective of this study was to prepare a clinically-oriented classification system of salivary gland cytology, and to apply this classification system to 1175 patients with parotid gland tumors who were treated at our department and in whom the final pathological diagnosis had been confirmed. Based on the results of this study, we discuss the advantages and limitations of a new classification system.

## Patients and methods

### Patients

Study subjects included 1175 patients with parotid gland tumors with confirmed histology who underwent surgery at the Department of Otolaryngology-Head and Neck Surgery at Osaka Medical College during the past 20 years from September 1999 to December 2019 (Table 1). In terms of histology, 981 patients had a benign tumor and 194 patients had a malignant tumor (low/intermediate-grade malignancy: 113 patients, high-grade malignancy: 81 patients). The most common benign parotid gland tumor was pleomorphic adenoma (605 patients), followed by Warthin tumor (225 patients), and basal cell adenoma (47 patients). For malignant parotid gland tumor, mucoepidermoid carcinoma was most common (51 patients) (low/intermediate grade: 26 patients, high grade: 25 patients), followed by carcinoma ex pleomorphic adenoma (29 patients) (low/intermediate grade: 11 patients, high grade: 18 patients), adenoid cystic carcinoma (22 patients) (low/intermediate: 17 patients, high grade: 5 patients), and salivary duct carcinoma (18 patients) (all patients were high grade). Metastatic lymph nodes within the parotid gland and malignant lymphoma were not included in this study.

**Table 1:** Clinical data and final histopathological results of patients with salivary gland tumors

	Benign tumor (*n* = 981)	Malignant tumor (*n* = 194)	
Age (mean)	12-90 (52.6)	14-85(54.4)	
Sex(male:female)	437:544	107:87	
Histological type			
Benign tumor			
Pleomorphic adenoma	605		
Warthin tumor	225		
Basal cell adenoma	47		
Others	104		
Malignant tumor			
Mucoepidermoid carcinoma	51	Basal cell adenocarcinoma	11
Carcinoma ex pleomorphic adenoma	29	Epithelial-myoepithelial carcinoma	11
Adenoid cystic carcinoma	22	Squamous cell carcinoma	9
Salivary duct carcinoma	18	Adenocarcinoma,not otherwise specified	6
Secretory carcinoma	16	Myoepithelial carcinoma	4
Acinic cell carcinoma	12	Others	5

### Fine-needle aspiration cytology (FNAC)

FNAC was conducted preoperatively for all 1175 cases, and it was performed predominantly by ENT specialists. FNAC was performed under ultrasound guidance using a 21- or 22-gauge needle with the patient in a supine position. Two to three aspirations were obtained with the freehand technique. Repeat FNAC was not performed even when the amount of specimen was deemed inadequate. Smears were directly stained using the Papanicolaou method, and cytopathological examinations were performed by experienced cytologists (co-authors). The preoperative cytology results obtained from FNAC were compared with the final histopathologic diagnoses made after surgical excision.

### Classification of cytopathology: Osaka Medical College (OMC) classification

FNAC diagnoses were classified into the following 11 categories (6 principal categories and 5 subcategories) (Table 2). “1–1 Inadequate”: this refers to samples judged as not suitable for cytologic assessment. “1–2 Cyst contents”: samples having only cystic fluid, without epithelial components. “2 Non-neoplastic”: this category includes inflammation, metaplasia, and other reactive changes deficient of tumorigenicity. “3 Atypia of undetermined significance (AUS)”: samples that were insufficient for a neoplastic lesion: a neoplastic process cannot be excluded after examination of all the cellular material. Samples diagnosed as benign tumor were classified as Category 4 and further classified into those in which histology was identified [“4–1 Benign tumor (histology confirmed)”] or not [“4–2 Benign tumor (histology not confirmed.)”]. Samples that were diagnosed as a tumor but for which histology could not be identified and malignancy was not ruled out were classified as “5 Salivary gland neoplasm of uncertain malignant potential (SUMP)”. Category 6 encompassed samples for which malignant tumor was suspected or diagnosed. This category was further subclassified into the following 4 groups: those in which malignancy was strongly suspected, but could not be confirmed (6–1), those which were diagnosed as malignant but the malignancy grade and histology could not be determined (6–2), those which were diagnosed as malignant and the malignancy grade could be determined (6–3), and those which were diagnosed as malignant and both malignancy grade and histology could be determined (6–4).

**Table 2:** The Osaka Medical College (OMC) categorical system for reporting salivary gland cytopathology categories

1-1 Inadequate
1-2 Cyst contents
2 Non-neoplastic
3 Atypia of undetermined significance (AUS)
4-1 Benign tumor (histology confirmed)
4-2 Benign tumor (histology unconfirmed)
5 Uncertain malignant potential (SUMP)
6-1 Suspicious for malignancy
6-2 Malignant (grade/histology unconfirmed)
6-3 Malignant (grade confirmed)
6-4 Malignant (grade/histology confirmed)

### Methods

Based on the final pathological diagnosis (benign or malignant tumor), the FNAC diagnosis (i.e., OMC Classification System) was evaluated in 981 benign tumors and 194 malignant tumors. Malignant tumors were further classified into low/intermediate-grade malignancy (113 patients) and high-grade malignancy (81 patients). The risk of malignancy (ROM) was also calculated for each FNAC diagnosis.

Next, the usefulness of the FNAC diagnosis (OMC Classification System) was evaluated based on the final pathological diagnosis (histology). If the final pathological diagnosis was malignant, the evaluation was conducted for mucoepidermoid carcinoma, carcinoma ex pleomorphic adenoma, adenoid cystic carcinoma, salivary duct carcinoma, secretory carcinoma, acinic cell carcinoma, basal cell adenocarcinoma, epithelial − myoepithelial carcinoma, squamous cell carcinoma, adenocarcinoma not otherwise specified, and myoepithelial carcinoma. If the final diagnosis was benign, the evaluation was conducted for pleomorphic adenoma, Warthin tumor, and basal cell adenoma. Finally, mucoepidermoid carcinoma, carcinoma ex pleomorphic adenoma, and adenoid cystic carcinoma were stratified by grade of malignancy and evaluated.

Finally, the final diagnosis was reviewed based on the FNAC diagnosis. The final pathology diagnoses were reviewed for 729 patients who had benign histology (Category 4–1) on FNAC, 65 patients whose FNAC diagnosis was malignant histology/malignant grade (Category 6–4), and 87 patients in whom the grade of malignancy was diagnosed (Category 6–3 + 6–4).

## Results

### OMC classification for parotid benign and malignant tumors

The OMC classification was reviewed based on the final pathological diagnosis, where 194 patients had a malignant tumor (low/intermediate grade: 113 patients, high grade: 81 patients) and 981 patients had a benign tumor (Table 3).

**Table 3:** Cytopathological results for the Osaka Medical College (OMC) system for salivary gland tumors

	1-1 Inadequate	1-2 Cyst contents	2 Non-neoplastic	3 AUS	4-1 Benign: (histology confirmed)	4-2 Benign: (histology unconfirmed)	5 SUMP	6-1 Suspicious for malignancy	6-2 Malignant: (only)	6-3 Malignant: (grade confirmed)	6-4 Malignant: (histology/grade confirmed)
Malignant (*n* = 194) (113/81)	26 (18/8)	2 (2/0)	5 (4/1)	0	12 (9/3)	13 (13/0)	23 (15/8)	13 (10/3)	18 (7/11)	21 (6/15)	61 (29/32)
Benign (*n* = 981)	133	20	27	8	717	38	26	4	3	1	4
Total (%)	13.5	1.9	2.7	0.7	62.0	4.3	4.2	1.4	1.8	1.9	5.5
Risk of malignancy (ROM) (%)	16.4	9.1	15.6	0.0	1.6	25.5	46.9	76.5	85.7	95.5	93.8

The number in parentheses indicates each case by histological grade (low or intermediate/high grade)

*AUS* atypia of undetermined significance, *SUMP* salivary gland neoplasm of uncertain malignant potential

Among those patients with a parotid gland tumor (1175 patients), there were 729 patients (62.0%) who were diagnosed as benign and had the histological type determined (Category 4–1). In addition, there were 65 patients (5.5%) who had a malignant tumor with histological type determined (Category 6–4). Overall, histological types were determined for 67.6% of patients.

Among those 194 patients with a malignant tumor, 113 patients (58.2%) were diagnosed as “malignant” using the OMC classification (Category 6). The grade of malignancy (Category 6–3 + 6–4) was assigned to 82 patients (42.3%). Both histology and grade of malignancy (Category 6–4) were determined for 61 patients (31.4%). The remaining malignant tumors were classified as SUMP in 23 patients (11.7%), “benign” (Category 4) in 25 patients (12.9%), and inadequate in 26 patients (13.4%). For low/intermediate-grade carcinoma (113 patients), patients who were determined as “malignant” (Category 6) and in whom “grade of malignancy” was determined (Category 6–3 + 6–4), and in whom “histology/grade of malignancy” (Category 6–4) were obtained were 46.0%, 31.0%, and 25.7%, respectively. However, for high-grade carcinoma (81 patients), this was 71.6%, 58.0%, and 39.5%, respectively.

Among those 981 patients with a benign tumor, 755 patients (77.0%) were diagnosed as “benign” (Category 4) and, of these, 717 patients (73.1%) had confirmed histology (Category 4–1). The remaining patients consisted of 26 patients (2.7%) categorized as SUMP, 12 patients (1.2%) categorized as “malignant” (Category 6), and 133 patients (13.6%) had an inadequate sample.

The ROM of 6–1, 6–2, 6–3, and 6–4 was 76.5%, 85.7%, 95.5% and 93.8%, respectively. Among those 125 patients who were diagnosed as “malignant” (Category 6) by cytology, 113 patients (90.4%) were also confirmed malignant on final pathology, and 46.9% of the patients diagnosed as SUMP and 25.5% diagnosed as benign (histology unconfirmed) were malignant at final pathology. On the other hand, 98.4% of patients who were diagnosed as benign histology (Category 4–1) were benign at final pathology. Cyst contents (Category 2) were diagnosed by final pathology as 20 cases with benign, 2 cases with malignant tumors. Two cases in malignant tumors were diagnosed as mucoepidermoid carcinoma (low grade) and acinic cell carcinoma. 20 cases in benign tumors were diagnosed as pleomorphic adenoma with three cases, Warthin tumor with two cases, and the others with salivary gland cyst (mucous cyst, salivary duct cyst, and lymphoepithelial cyst).

### OMC classification for each final histological type

The FNAC diagnosis (OMC classification) was evaluated based on the final pathological diagnosis (Table 4).

**Table 4:** Cytopathological results for the Osaka Medical College (OMC) system for each final pathology

	1-1 Inadequate	1-2 Cyst contents	2 Non-neoplastic	3 AUS	4-1 Benign: (histology confirmed)	4-2 Benign: (histology unconfirmed)	5 SUMP	6-1 Suspicious for malignancy	6-2 Malignant: (only)	6-3 Malignant: (grade confirmed)	6-4 Malignant: (histology/grade confirmed)	Total
Malignant tumor												
Mucoepidermoid carcinoma	8 (6/2)	1 (1/0)	3 (2/1)		2 (1/1)	3 (3/0)	7 (4/3)	4 (3/1)	3 (1/2)	3 (1/2)	17 (4/13)	51
Carcinoma ex pleomorphic adenoma	2 (1/1)				4 (2/2)	3 (3/0)	3 (0/3)	1 (0/1)	6 (2/4)	4 (1/3)	6 (2/4)	29
Adenoid cystic carcinoma	3 (2/1)				1 (1/0)	2 (2/0)	3 (3/0)	1 (1/0)	3 (0/3)		9 (8/1)	22
Salivary duct carcinoma	1						1		1	8	7	18
Secretory carcinoma		1	1		2	1		3		1	7	16
Acinic cell carcinoma					1		1	2	1	1	6	12
Basal cell adenocarcinoma	2					2	4	1		1	1	11
Epithelial-myoepithelial carcinoma	5				2		3				1	11
Squamous cell carcinoma	2						1		1	1	4	9
Adenocarcinoma, not otherwise specified	1							1		1	3	6
Myoepithelial carcinoma	1					2			1			4
Benign tumor												
Pleomorphic adenoma	40	3	7	3	515	16	12	2	3	1	3	605
Warthin tumor	34	2	10	3	166	6	3	1				225
Basal cell adenoma	11				21	11	3				1	47

The number in parentheses indicates each case by histological grade (low- or intermediate/high grade)

*AUS* atypia of undetermined significance, *SUMP* salivary gland neoplasm of uncertain malignant potential

For 51 patients with mucoepidermoid carcinoma (low/intermediate grade: 26 patients, high grade: 25 patients), 27 patients (52.9%) were diagnosed as “malignant” (Category 6). By the grade of malignancy, 34.6% of patients with a low/intermediate grade and 72.0% with a high grade were diagnosed as “malignant”. Grade of malignancy (Category 6–3 + 6–4) was determined in 19.2% of low/intermediate grade and 60.0% of high grade. For 29 patients with carcinoma ex pleomorphic adenoma (low/intermediate grade: 11 patients, high grade: 18 patients), 17 patients (58.6%) were diagnosed as “malignant” (Category 6). By grade of malignancy, the “malignant” category was assigned in 45.5% of those with low/intermediate grade and 66.7% of high grade. The grade of malignancy (Category 6–3 + 6–4) was assigned to 27.3% in low/intermediate grade and 38.9% in high grade. Other histology types are shown in Table 4.

The FNAC diagnosis (OMC classification) was reviewed for patients whose final pathological diagnosis was a benign tumor, including pleomorphic adenoma (605 patients), Warthin tumor (225 patients), or basal cell adenoma (47 patients). The percentage of patients in whom benign histology was diagnosed by OMC classification (Category 4–1) was 85.1%, 73.8%, and 44.9%, respectively. The percentages of these patients who were diagnosed as “malignant” (Category 6) by FNAC were 1.5%, 0.4%, and 2.1%, respectively.

### Final pathology for cases with histology/grade confirmed by FNAC

The final pathological diagnosis was reviewed for those 729 patients who were diagnosed as benign by FNAC (Category 4–1) (Table 5). The histopathology according to the FNAC diagnosis of the 729 patients were as follows: pleomorphic adenoma (539 patients), Warthin tumor (179 patients), and basal cell adenoma (8 patients). The final diagnosis of patients who were diagnosed as pleomorphic adenoma by FNAC was as follows: pleomorphic adenoma (accurate histology), 508 patients (94.2%); other benign histology, 22 patients (4.1%); and malignancy, 9 patients (1.7%). The final diagnosis of patients who were determined to have Warthin tumor by FNAC were as follows: Warthin tumor (accurate histology), 164 patients (91.6%); other benign histology, 12 patients (6.7%); and malignancy, 3 patients (1.7%). Final pathology results of patients who were diagnosed as basal cell adenoma were as follows: correct histology, 7 patients, and other benign histology, 1 patient. Overall, an accurate FNAC histologic diagnosis was made in 681 of 729 patients (93.4%).

**Table 5:** Detailed results of final pathology for Category 4–1 (benign tumor, histology established)

					FNAC diagnosis (Category 4–1; *n* = 729)		
				Pleomorphic adenoma 539	Warthin tumor 179	Basal cell adenoma 8	Others 3
Final pathology							
Benign: histopathology correct			681(93.4%)	508	164	7	2
Benign: histopathology incorrect			36 (4.9%)	22	12	1	1
Malignant			12 (1.6%)	9	3	0	0

*FNAC* fine-needle aspiration cytology

The final diagnosis of those 65 patients (Category 6–4) who were diagnosed as malignant by FNAC (confirmed by both histology and grade) were reviewed (Table 6). The main histopathology of the 65 patients (FNAC diagnosis) were as follows: Adenoid cystic carcinoma, 20 patients; acinic cell carcinoma, 18 patients; mucoepidermoid carcinoma, 17 patients; and squamous cell carcinoma, 7 patients. When compared with the final pathological diagnosis, the FNAC diagnosis was accurate (both histology and grade of malignancy) in 40 patients (61.5%), and accuracy of grade of malignancy was only 18 patients (27.7%). Therefore, the accurate diagnosis of the grade of malignancy by FNAC was 89.2%.

**Table 6:** Detailed results of final pathology for Category 6–4 (malignant, both grade and histology established)

			FNAC diagnosis (Category 6–4; *n* = 65)					
		MEC	Ca ex pleo	AdCC	ACC	SCC	BCC	EMC
Low, intermediate-grade/high-grade	36/29	4/13	0/1	12/8	18/0	0/7	1/0	1/0
Final pathology								
Malignant(histology/grade) correct	40(61.5%) 23/17	3/6	0/1	8/3	12/0	0/7	0	0
Malignant(grade) correct	18(27.7%) 6/12	1/7	0/0	2/5	2/0	0	0	1/0
Malignant(only) correct	3(4.6%) 3/0	0/0	0/0	1/0	2/0	0	0	0
Benign	4(6.2%) 4/0			1/0	2/0		1/0	

*ACC* acinic cell carcinoma, *AdCC* adenoid cystic carcinoma, *BCC* basal cell adenocarcinoma, *Ca ex pleo* carcinoma ex pleomorphic adenoma, *EMC* epithelial − myoepithelial carcinoma, *FNAC* fine-needle aspiration cytology, *MEC* mucoepidermoid carcinoma, *SCC* squamous cell carcinoma, *SDC* salivary duct carcinoma

The final diagnosis of those 87 patients who were diagnosed as malignant (grade of malignancy confirmed) by FNAC (Category 6–3 and 6–4) were reviewed (Table 7). Of these 87 patients, there were 77 patients (88.5%) whose grade of malignancy was confirmed as accurate; 43/44 patients who had high grade and 34/43 patients who had low/intermediate malignancy.

**Table 7:** Detailed results of final pathology for Category 6–4 and 6–3 (malignant; grade established)

		FNAC diagnosis (Category 6–3, 6–4; *n* = 87)	
		Low/intermediate-grade 43	High-grade 44
Final pathology			
Malignant(grade) correct	77(88.5%)	34	43
Malignant(only) correct	5(5.7%)	4	1
Benign	5(5.7%)	5	0

*FNAC* fine-needle aspiration cytology

## Discussion

FNAC is an effective method for the diagnosis of salivary gland tumors. However, its diagnostic performance is affected by differences in techniques, procedures, and the capability of the cytopathologist. The classification of cytological diagnoses is also an important issue to consider. Ultimately, expectations for the utility of an FNAC diagnosis should be considered. For salivary gland tumors, it must be decided if the diagnosis of benign or malignant is sufficient, or whether histology must be determined for an accurate diagnosis. Furthermore, for malignant tumors, grade of malignancy is closely related to the prognosis and is essential information.

In 2018, the Milan system was proposed as a new cytology classification for salivary gland tumors [5]. The system classified the diagnosis into seven categories. In 2016, Bajwa et al. [8] revised the Milan cytology classification and reported the Sal classification. Kilavuz et al. [9] evaluated the Sal classification in 312 patients with salivary gland tumors and concluded that this classification was useful. Several validation studies have been conducted for the Milan system, as well, and its effectiveness was reported [10–12]. Wang et al. [13] and Griffith et al. [14] have also proposed cytology classification systems for salivary gland tumors. However, none of these cytology classifications for the salivary gland consider histology and grade of malignancy. ROM and optional reports, including histopathology and the grade of malignancy is left to the individual pathologist or laboratory in benign and malignant tumors in the Milan System. In our system, we have specified histopathology and the grade of malignancy in the category. Only Mazzola et al. [15] has reported a classification system of malignant tumors based on low- and high-grade malignancy, providing a malignancy diagnosis rate of 86.4% and 97.0%, respectively. However, their study included 55 patients with malignant salivary gland tumors and 64 patients with malignant nonsalivary tumor (among them, 55 had squamous cell carcinoma). Therefore, it is difficult to consider their study as being solely for salivary gland tumors. Moreover, only 22 patients with low-grade carcinoma were included.

FNAC is the only direct diagnostic method for the preoperative diagnosis of histology and grade of malignancy for salivary gland tumors. The preoperative diagnosis of histology and grade is useful when determining treatment strategy. It is well known that the prognosis for salivary gland carcinoma is markedly different depending on grade of malignancy [7]. Accordingly, treatment strategy and surgical approaches, including the extent of resection and preservation of the facial nerve, vary based on grade of malignancy. In terms of histology of malignant tumors, adenoid cystic carcinoma is known to be associated with perineural invasion [16] and, thus, extended resection is desirable. Although, for the benign tumor pleomorphic adenoma, enucleation is contraindicated. If the histology shows a Warthin tumor, course observation is an option as it does not progress to malignancy [6, 17]. Thus, the conventional classification, which lacks consideration of grade and histology, is not entirely satisfactory for clinicians. Therefore, we developed the OMC classification system, which incorporates grade of malignancy and histology.

To verify the appropriateness of the OMC classification, it was first necessary to evaluate its reliability in diagnosing grade of malignancy and histology based on FNAC. The percentage of patients who had a benign tumor and had histological diagnosis (Category 4–1) was 62.0%. Among these patients, the diagnosis of 93.4% was consistent with the final pathological diagnosis. The percentage of patients who had a malignant histology and grade (Category 6–4) was 5.5%. Among these patients, the diagnosis of 61.5% was consistent with the final pathological diagnosis. In short, histology was diagnosed for 67.6% of overall patients based on FNAC, and the correct diagnosis rate was high. With regard to grade of malignancy, 7.4% of patients also had the grade of malignancy determined (Category 6–3 + 6–4). Among them, the diagnosis of 88.5% of patients was consistent with the final pathological diagnosis; more specifically, the correct diagnosis rate in high-grade carcinoma was high as well. Thus, incorporation of histology and grade of malignancy into the FNAC classification was found feasible and appropriate.

The use of FNAC to determine both grade of malignancy and histology would be ideal if possible. However, as emphasized in the conventional classification system, the differentiation between benign versus malignant tumors is the minimum requirement of the FNAC diagnosis. Hence, understanding the ROM in each category of the cytology classification system is clinically very important. In addition, it validates a given classification system. According to a review of the Milan system, the following percentage of patients, classified as below, were found malignant by the final pathology: 25% in nondiagnostic, 10% in non-neoplastic, 20% in AUS, 35% in SUMP, 80% in suspicious for malignancy, and ≥ 90% in malignant [18]. In the present study, the ROM was 16% in nondiagnostic, 16% in non-neoplastic, 47% in SUMP, 77% in suspicious for malignancy, and 93% in malignant and was comparable to the result of the Milan system. As for benign tumors, ROM in category 4–1 (Benign: histology confirmed) accounted for only 1.6%, while that in category 4–2 (Benign: histology unconfirmed) reached 25.5%. The ROM for category 4–2 was high compared with the Milan system. The cases which were “reactive and reparative atypia indefinite for a neoplasm” and “low cellularity specimens suggestive of, but not diagnostic of a neoplasm” were classified into AUS according to cytologic criteria under the Milan system. In these cases, a discussion between clinicians and cytopathologists as for their categorization often took place. When clinicians diagnosed them as tumorous lesion by clinical data, yet cytopathologists sometimes did not categorize these cases into category 3 (AUS), but into category 4–2 and, thus, the number of category 4–2 was increased in this study. In fact, parotid tumors are often easily distinguishable tumors and nontumor lesions clinically. In our classification system, it is our policy to try to classify cases into more detailed categories, such as category-4 and -6, as far as possible. Accordingly, it is considered that this new classification system was appropriate in terms of differentiating between benign versus malignancy.

## Conclusions

Conventional cytology classification systems for salivary gland tumors do not include histology and grade of malignancy. However, the preoperative diagnosis of histology and grade of malignancy is beneficial when determining the treatment strategy. Therefore, we developed a new cytology classification system that incorporates both histology and grade of malignancy (the OMC classification). We then applied the OMC classification to 1175 patients who underwent surgery at our hospital and for whom a final pathological diagnosis was confirmed (malignant tumor: 194 patients, benign tumor: 981 patients). Based on FNAC, 729 patients (62.0%) were diagnosed with benign histology, and 65 patients (5.5%) had malignant histology/grade. When these results were compared with the final histopathology, the correct diagnosis rate was 93.4% in benign and 61.5% in malignant tumors. For the malignancy grade alone in FNAC, the correct diagnosis rate was 88.5%. Accordingly, it is feasible and appropriate to include histology and grade of malignancy in a cytology classification, and such a classification is considered clinically useful.
